# Energy Efficient Pupil Tracking Based on Rule Distillation of Cascade Regression Forest

**DOI:** 10.3390/s20185141

**Published:** 2020-09-09

**Authors:** Sangwon Kim, Mira Jeong, Byoung Chul Ko

**Affiliations:** Department of Computer Engineering, Keimyung University, Daegu 42601, Korea; swkim@stu.kmu.ac.kr (S.K.); mystroll@kmu.ac.kr (M.J.)

**Keywords:** pupil tracking, on-device machine learning, cascade regression forest, rule distillation, transparent model

## Abstract

As the demand for human-friendly computing increases, research on pupil tracking to facilitate human–machine interactions (HCIs) is being actively conducted. Several successful pupil tracking approaches have been developed using images and a deep neural network (DNN). However, common DNN-based methods not only require tremendous computing power and energy consumption for learning and prediction; they also have a demerit in that an interpretation is impossible because a black-box model with an unknown prediction process is applied. In this study, we propose a lightweight pupil tracking algorithm for on-device machine learning (ML) using a fast and accurate cascade deep regression forest (RF) instead of a DNN. Pupil estimation is applied in a coarse-to-fine manner in a layer-by-layer RF structure, and each RF is simplified using the proposed rule distillation algorithm for removing unimportant rules constituting the RF. The goal of the proposed algorithm is to produce a more transparent and adoptable model for application to on-device ML systems, while maintaining a precise pupil tracking performance. Our proposed method experimentally achieves an outstanding speed, a reduction in the number of parameters, and a better pupil tracking performance compared to several other state-of-the-art methods using only a CPU.

## 1. Introduction

Human gaze perception during a social interaction is an important non-verbal factor facilitating communication between people. In the field of machine learning (ML) in particular, human gaze perception is essential as an interface for a human–machine or human–computer interaction (HCI). Human gaze perception during an HCI can be identified through eye pupil tracking. Therefore, for accurate gaze perception, the core technology, pupil detection, and tracking, must be preceded. Human pupil tracking is a technique used to track the position and direction of the pupil center based on the pupil movement in both eyes, and is used in various applications along with various cognitive HCI devices. For example, pupil tracking technology is rapidly emerging as a core technology of HCI, particularly in virtual reality (VR) and augmented reality (AR), because it allows convenient device control without a specific controller in various devices. It can also be used as a component technology for an advanced driver assistant system (ADAS) that can detect the driver’s inattentive behavior or drowsiness within a smart vehicle field in advance. Biometrics, the identification of consumer interests, and advertisements for marketing analysis are also important applications of pupil tracking. Infrared camera based pupil tracking methods, which are widely used in gaze perception, focus on an estimation of the pupil position by analyzing the difference in reflectance between the iris and pupil through infrared illumination and a near-infrared camera. However, the use of an infrared camera requires additional LED illuminators and infrared camera devices, and has a problem of a poor performance when users are wearing glasses.

Because pupil tracking applied to HCI requires an extremely precise location tracking, the use of a deep neural network (DNN) has emerged as a general approach to ML, yielding state-of-the-art results in pupil tracking studies with the availability of big data. The DNN-based pupil tracking method has improved the accuracy more than traditional that of ML-based approaches using handcrafted features; however, as the network structure deepens and becomes more complicated in terms of a performance improvement, the requirements for the system resources are increasing, and it is difficult to operate an on-device ML system in advance. High-performance ML systems have commonly been carried out by sending information collected from mobile devices to a cloud server for analysis and sending information back to the device. In contrast, on-device ML processes information inside the smart device itself without going through a remote cloud server, enabling faster work with a low latency, fewer data security concerns, and support for poor network connectivity. Therefore, it allows the accessibility to a large number of applications, such as gaming and HCI [1]. On-device ML (on-device artificial intelligence (AI) and ML are often used interchangeably; in the paper, we use the term on-device ML) is closely related to interpretable machine learning (IML) and energy-efficient ML. In terms of the ML model, interpretable means that the model itself is not complicated and transparent, so that the decision making process can be understood. In other words, ML algorithms that can operate on smart devices must be made energy-efficient through a lightweight model and must be interpretable for analyzing the reason for errors or consequences of the operational process.

The goal of IML is to create a suite of ML techniques that produce more explainable models, while maintaining a high learning performance [2]. However, to create an IML model, we need to convert a complex black-box model into an interpretable (or transparent) white-box model, or create an additional model to explain the results of an existing black-box model. Although an IML model based on a black-box is easy to implement using an existing DNN structure, the entire system becomes more complicated because an additional explanatory model has to be created to explain the black-box model.

An energy-efficient ML that can operate at low power even in low-spec systems has been continuously applied in connection with on-device IML studies. In a resource-constrained environment, it is improper to only consider the accuracy of the model, and thus system designers must carefully balance power, energy, and size along with performance.

**Figure 1:**
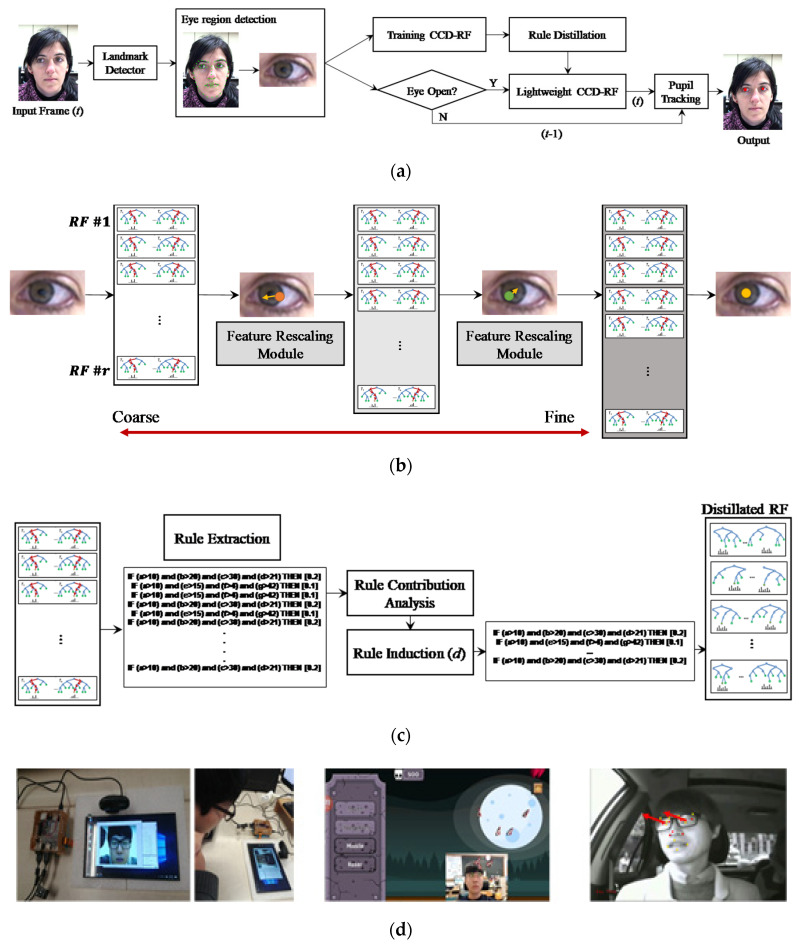
(**a**) Overall procedures of the proposed pupil tracking approach. (**b**) After training CCD-RF for pupil position, if the eye is opened, the pupil position is predicted sequentially using a coarse-to-fine manner of a trained CCD-RF. (**c**) The pupil tracking is applied based on the spatial relation between the pupil positions of the previous and current frames. The proposed rule distillation is applied to a trained CCD-RF and only important rules are remained per each RF. (**d**) Examples of various real-time applications where distillated CCD-RF can be applied (left: HCI tool for embedded device, middle: smartphone game, right: driver attention monitoring).

Figure 1a shows the overall procedure for the pupil tracking approach proposed in this paper. In this study, the motivations for each component of the proposed algorithm is as follows. First, to develop a real-time pupil tracker that operates on a low-spec device, we adopt a cascade coarse-to-fine deep regression forest (CCD-RF) instead of a black-box DNN structure (Figure 1b). Although the RF has the advantage of a simple structure and fast operation compared to a DNN, a CCD-RF is still consisted of complex rules and requires a large number of parameters and operations. Therefore, second, to remove less important rules for each tree consisting of the RF, we apply a new rule distillation algorithm to CCD-RF as shown in Figure 1c. Third, to accurately detect the pupil in image sequences while excluding falsely detected pupil positions, pupils are tracked based on consistency check between two eyes. The pupil tracking approach designed through these motivations can be applied to low-power devices because it is not only interpretable by reducing the rules of CCD-RF, but also energy-efficient due to reduced computation. Figure 1d shows various embedded systems and application examples to which the proposed method can be applied.

## 2. Related Work

The goal of this study is to design a lightweight pupil tracking model that is interpretable and energy efficient for application to an on-device AI. Therefore, we divide the related studies into the research trend of IML and pupil-tracking based on a light AI model.

### 2.1. Interpretable ML with Energy Efficiency

IML explores and investigates how ML models are created or complemented, allowing humans to access and interpret the results of internal logic and algorithms, allowing humans to understand these processes [3]. IML can be divided into a transparent model revealing how it functions, and a post-hoc explanation that describes why a black-box model behaves depending on the model’s explainability [4]. However, the post-hoc model cannot reduce the size of the model because it trains a separate explainable model while maintaining a black-box model such as a DNN. Therefore, in this study, we focus on a transparent model through various IML approaches in terms of the model interpretability and energy efficiency.

In most transparent models [5,6], an interpretable and simple reconstruction has been focused upon, unlike in inherently complex models. The surrogate model approach uses an interpretable model as a substitute for describing a complex model, for example, training a black box model as the training data and training a white box model that can be interpreted as the training data. Dasari et al. [5] explored a global surrogate RF model by hyper-parameter tuning to improve understanding of the design space.

A Bayesian rule list (BRL) [6] is a decision list consisting of a series of IF-THEN statements. BRLs discretize a high-dimensional, multivariate feature space into a series of simple, readily interpretable decision statements. A scalable BRL [7] is an iteratively optimized decision list with a strikingly practical balance among its accuracy, interpretability, and computational speed, resulting in an interpretable and practical computational time if fewer optimal decision rules are selected. However, because the IF-THEN rules of BRL focus on classification and almost completely neglect a regression, this algorithm is unsuitable for gaze tracking. Ensemble learning based on an RF or gradient boosting is a popular method of ML classification and regression because it can handle an overfitting and reduce the variance compared to a single decision tree or a linear or complex non-linear function.

A scalable end-to-end tree boosting system called XGBoost [8] is widely used by data scientists to achieve state-of-the-art results for numerous ML challenges because it applies a more regularized model formalization to control an over-fitting, which gives it a better performance. XGBoost achieves a good performance and parallel operation; however, it requires a lengthy amount of time to learn a large number of parameters.

Interpreted and simplified LMRF (iLMRF) [9] is a newly proposed model interpretation and simplification scheme that eliminates unnecessary tree rules in multi-layer random forests based on their feature contributions. Although iLMRF is a simplified but dense model with fewer parameters and rules than the original model, it is not a fully white model because it still contains numerous rules and feature parameters and is optimized to deal with classification problems.

### 2.2. Pupil Tracking

In the field of pupil tracking, new ML studies on a lighter DNN have been conducted to achieve a fast processing for an on-device system.

First, using conventional image processing techniques, Levinshtein et al. [10] proposed a cascaded regressor for eye center detection using a facial feature alignment and circle fitting. By extension, using typical ML-based methods, Santini et al. [11] proposed robust and fast pupil tracking method based on pure edge detection as well as segmentation to sort out candidates. In addition, from the preprocessed candidate footages, the proposed confidence measurement is performed with three given metrics: ellipse aspect ratio, angular edge spread, and ellipse outline contrast. Zhang et al. [12] proposed a selective oriented gradient filter to remove strong gradients from the eyebrows, eye corners, and shadows to achieve an accurate and real-time pupil detection from an image; thus, allowing a coarse-to-fine, global-to-regional scheme. Xia et al. [13] employed an isophote curvature method to an eye region to obtain several candidate points of the eye center and used a supervised descent regressor based on a gradient approach to obtain the rough location of the eye region and the eye centers. In addition, Ince et al. [14] proposed a low-cost pupil center localization algorithm based on the maximized integral voting of the candidate kernels of a circular hollow kernel. An estimation of the pupil center is employed by applying a rule-based schema for each pixel over the eye sockets. George et al. [15] proposed a two-stage algorithm for iris center localization consisting of the base location of a coarse iris by applying a convolutional filters and a refining stage using boundary tracing and ellipse fitting.

In terms of DNN-based pupil tracking methods, He et al. [1] proposed the use of on-device few-shot personalization methods for a 2D pupil estimation based on an unsupervised personalization method, which uses only unlabeled facial images to improve the accuracy of the gaze estimation. However, because this method still depends on a heavy end-to-end convolutional neural network (CNN) model, it is difficult to interpret the model and requires a greater reduction in the number of operations for on-device use in real-time. Xia et al. [16] adapted a fully convolutional neural network (CNN) into a shallow structure with a large kernel convolutional block and transferred the performance from semantic segmentation to an eye center localization task through fine-tuning. Other methods have recently attempted to apply a generative adversarial network (GAN) to gaze perception. The purpose of using a GAN for gaze perception is to generate synthetic training data to improve the 3D pupil estimation accuracy and support a user-specific adaptation. Gou et al. [17] proposed the use of a parallel imaging step built upon a GAN to generate adversarial synthetic images for training purposes. For the pupil detection, a coarse-to-fine framework is used based on shape-augmented cascade regression models learned from adversarial synthetic images. Choi et al. [18] used heterogeneous GANs and CNN models depending on whether people in the images are wearing glasses. For the user-specific pupil adaptation, Yu et al. [19] generated additional training samples through the synthesis of gaze-redirected eye images from existing reference samples. Similar to [19,20] also proposed a framework for a few-shot adaptive gaze estimation for the learning of person-specific gaze networks by applying very few calibration samples. However, these GAN user-specific gaze adaptation approaches primarily use a transforming GAN and encoder-decoder architectures, which require fine-tuning to adapt the model to a new subject, and thus user-specificity and personalization are computationally intensive, requiring a large amount of calibration data, and cannot be run on low-spec devices [1].

As indicated in the above review, recent studies on pupil perception and tracking have mostly been based on a CNN or GAN, and have mainly focused on improving the personalization and 3D pupil estimation accuracy. However, the simplification of the pupil tracking model presents several benefits, such as an improved model interpretability, energy efficiency from low operations, and a low latency during tracking without a loss in accuracy. As a result, lightweight transparent models can be applicable to a large number of games and HCI applications.

### 2.3. Contribution of This Work

In this study, we propose a lightweight CCD-RF for interpretable and simplified pupil tracking in a 2D gaze estimation. This method achieves a better accuracy than prior DNN-based approaches with significantly reduced parameters and operations. The main contributions are as follows:

- cascade pupil estimation technique consisting of a fast and accurate cascade deep RF instead of a DNN-based pupil estimation;
- estimation of the pupil location per layer in a coarse-to-fine manner and a refinement of the pupil area from the estimated location and eye area of the previous layer;
- RF simplification (rule distillation) removes low-importance rules according to the feature importance during the process of reconstructing a rule set;
- the rule distillation proves that the performance is not degraded even if numerous rules and parameters are eliminated;
- pupil consistency checking robust to fast movements of the face and maintain an accurate pupil tracking;
- proposed model not only consumes much less energy than other DNN-based methods, it also requires less computing power to enable a real-time operation on a CPU; and
- various experiments to establish the significantly faster processing time (approximately 1.55-fold speed up in gaze inference), lighter parameters (approximately 48-fold reduction in the number of parameters), and energy consumption efficiency (approximately 1.76-fold reduction in energy consumption based on a lightweight CCD-RF).

The remainder of this paper is organized as follows. In Section 3, we describe the rule distillation of a cascade RF for estimation of the eye pupil. In Section 4, we introduce the details of the proposed simplified CCD-RF with a tracking. In Section 5, we present experiments demonstrating the accuracy and energy efficiency. Finally, some concluding remarks and future study are presented in Section 6.

## 3. Rule Distillation of Cascade

State-of-the-art DNN-based studies [20,21] follow similar steps in a single network using a layer-by-layer structure and an end-to-end learning paradigm. Although a DNN-based pupil estimation has achieved outstanding results, it has certain limitations, such as too many hyper-parameters, a reliance on black-box training, high processing costs, and the requirement of vast amounts of training data. Therefore, to overcome these disadvantages, we facilitate our understanding of an RF specifically using a simplification technique through a rule analysis based on the feature importance. Because a regression tree of an RF splits a complex nonlinear regression problem into a set of smaller problems that can be more easily handled using simpler models, it is more interpretable than other non-linear ensemble approaches.

The rule distillation of CCD-RF is achieved by eliminating nontrivial rules and maintaining highly important rules, based on the results of a contribution analysis for all possible rules. Unlike in a previous study [9], the primary contribution of this research is to build a simplified interpretable regression model using a contribution metric re-defined to be compatible with a regression problem. In this section, we describe the rule distillation through an analysis of the features and rule importance that explicitly represent the decision making in the black box model from each regression tree.

Feature importance: The trained CCD-RF consists of a number of paths that can be represented by decision rules. Each path is made up of a set of nodes, which are the minimum learning unit, and each node applies a binary comparison for a given feature with the optimal conditions determined during the training phase. The decision process helps the node determine a split path according to the results of the binary comparison. The regression tree is trained by minimizing the least squares or minimum absolute error function as the form of an objective function. However, because the target variable is real and each independent variable is used to fit the regression model to the target variable, we use the least-squares error function (F) to find the split function that minimizes the errors. In a regression tree of an RF, the objective function of the *j*th split node is as follows:

(1) $$F_{j}=\sum_{S_{j}}{\left(∆d_{j}-{\bar{∆d}}_{j}\right)}^{2}-\sum_{i\epsilon \left\{L,R\right\}}\frac{\left|S_{j}^{i}\right|}{\left|S_{j}\right|}\left(\sum_{S_{j}^{i}}{\left(∆d_{j}-{\bar{∆d}}_{j}\right)}^{2}\right),$$

where ${\;S}_{j}$ indicates the set of training data arriving at node *j*. Here, *L* and *R* are the left and right split nodes, respectively, and ∆d={dx,dy} is the displacement vector from the eye corners to the pupil centers. In addition, ${\bar{∆d}}_{j}$ indicates the mean displacement vector of ∆d={dx,dy} for all training landmarks reaching the *j*th node.

This process continues until it reaches a leaf node by iterating over the optimal feature space while searching all nodes along the path. After training the regression tree, each leaf node predicts and stores a 2D displacement vector ∆d using training samples in the leaf node. To predict the displacement vector ∆d of each leaf node, we estimate a linear function that can represent the corresponding feature space well using all samples in the same leaf node. Among the various types of functions, such as the median, averaging, or non-linear function, a linear function is more interpretable than a non-linear function and less sensitive to outliers (noises) than the median and averaging functions.

In regression tree *t*, all nodes (not just the leaves) have an association with the upper and lower linked nodes. Each feature of the regression tree along the path is associated with every decision and contributes to the final outcome. Therefore, we can compute the final outcome in terms of the feature importance, which indicates how much a feature has helped improve the purity of all nodes.

The feature importance of the *j*th node (except the leaf node) $W^{t}\left(i,j\right)$ of the *i*th rule is calculated using the difference in regression vector between a parent node ${\bar{∆d}}_{j-1}$ and child node ${\bar{∆d}}_{j}$.

(2) $$W^{t}\left(i,j\right)=\left({\bar{∆d}}_{j-1}-{\bar{∆d}}_{j}\right)$$

Rule contribution analysis: The feature importance is a factor of how much a feature helps improve the purity of a feature space determined at a node. We extend this idea to move the domain from a single node to a path (rule) having multiple nodes. This requires a rule induction procedure that iteratively learns a single rule individually to create a decision set that includes the entire dataset [22]. To be specific, after a black-box CCD-RF is built, the individual rules from each RF should be iteratively induced and stored in the respective decision sets through a sequential covering [23].

A regression tree making up a regression forest consists of a set of nodes that can be interpreted by IF-THEN conditions. A path connecting multiple nodes is considered a rule that can be converged based on the conditions from the input provided to the root node to its corresponding leaf node. In this study, we modify a sequential covering to distill optimal rules based on the feature importance at every step during the tree traversal.

Rule distillation. At the tree level, the final rule contribution  C in a rule set L is estimated through a combination of the feature importance along the rule node path. After a rule set for all cascade RF layers is completed, to construct a reduced model, less important rules are eliminated according to the given rule contribution. Then, depending on the distillation rate *d* specified by the user, only rules with a high contribution remain in the rule set. The distillation rate can be applied to each rule uniformly, or it can achieve flexibility for each tree depending on the required accuracy. Algorithm 1 describes the overall procedure of rule distillation based on a rule contribution analysis for building an interpretable CCD-RF. The proposed method is applicable to each number of RFs that constitute multiple layers, and the redundancy that may exist during the decision process can be reduced by assigning a rule priority as a result of the feature contribution of each node.
**Algorithm 1:** Overall procedure of rule induction**Input:** t: trained tree, d: distillation rate **Output:** L{l}: Ordered Rule set consists of l layersInitialize rule set L=∅
**For** each l
**in**
Layer:
   **For** each r
**in**
RF:
      **For** each t
**in**
tree:
         Induce an *i*th rule ${ℒ}_{i}^{t}$ from a tree *t*         Calculate rule contribution C of a rule path p
         C(ℒit)=1p∑(i,j)∈pWt(i,j)
        (3)
         Append rule ${ℒ}_{i}^{t}\;$ and its $C\left({ℒ}_{i}^{t}\right)$ to $L{\left\{l\right\}}_{t}^{r}$
      **End for**
      Sort rules in $L{\left\{l\right\}}_{t}^{r}$ according to rule contribution      Eliminate some of rules with low contribution by *d*%
   **End for**
**End for**
**Return**
L

This procedure can be iterated until we extract all qualified rules that cover an RF. To begin with, extracting each decision rule from the given model means that the procedure should be performs on tree level of respective random forests on each layers. At tree level, we can induce as many rules ${ℒ}^{t}$ as the number of leaves in a specific tree *t*. Each atomic rules also can be represented as having decision paths, on each paths ${ℒ}_{i}^{t}$, we applied the given Equations (2) and (3) to calculate rule contribution $C\left({ℒ}_{i}^{t}\right)$ from each paths. Finally, rule elimination is applied on the set of rules with calculated rule contributions at each random forest level.

Table 1 shows the reconfiguring of the rules in a rule set. Through the proposed rule distillation procedure, induced rules are reordered according to the rule contributions of each tree.

## 4. Pupil Tracking and Consistency Check

**Figure 2:**
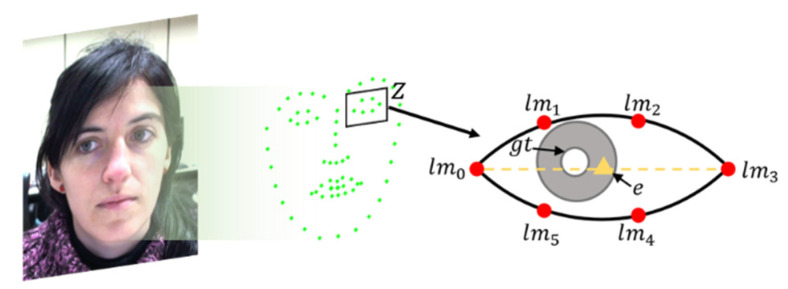
Six eye landmarks used for estimating the starting pupil position. The starting pupil position (e) of the eye is assigned to the middle location of both eye corners ($lm_{0}$ and $lm_{1}$), and the displacement (∆d) vector is computed using distance $L_{1}$ between a given ground truth (gt) and the predicted pupil position.

In this section, we present the details of the proposed regression method used to track the pupil within the detected eye regions. Eye regions are extracted by applying 12 coordinates in the detected face landmarks using Dlib [24], and we compose simple cropped gray-scale image patches as input features for both eyes ($Z_{1}\;\mathrm{and}\;Z_{2}$). Each observation $Z_{i}$ consists of feature vector $X_{i}$, and the coordinates at the center of the eye are then converted into displacements as a ground-truth value, as illustrated in Figure 2.

When eye image I and an initial pupil location $Z^{0}$ or previous pupil location $Z^{l-1}$ are given, the CCD-RF $RF^{l}\;$ of the *l*th layer computes $∆d_{l}=\left\{∆x_{l},∆y_{l}\right\}$ from the image patch. From the given information, the new pupil location $Z^{l}\;$ is updated in a cascade manner as follows:

(4) $$Z^{t}=Z^{l-1}+RF^{l}\left(I,Z^{l-1}\right),l=1,\ldots ,L.\;$$

When M training eye images with their ground-truth pupil location ${\left\{\left(I_{i},{\hat{Z}}_{i}\right)\right\}}_{i=1}^{M}$ are given, the RF at the *l* layer $RF^{l}\left(I,Z^{l-1}\right)$ are learned to minimize the sum of the pupil location residuals (offset error) on the training eye images using Equation (5).

(5) $$RF^{l}=arg\underset{CCD-RF}{\min}\overset{M}{\underset{i=1}{\sum }}\left\|{\hat{Z}}_{i}-\left(Z_{i}^{l-1}+RF\left(I_{i},Z_{i}^{l-1}\right)\right)\right\|$$

The predicted $∆d_{l}$ in the first layer is used to move to the pupil location of the next layer, and the new M training eye patches and their ground-truth are given based on the new pupil location. This process is repeated until *L* layers are sequentially generated. Rule distillation of the CCD-RF can be adjusted based on the user requirements after all *L* layers have been configured.

After training the CCD-RF of all layers, each leaf node predicts and stores a 2D displacement vector ∆d={∆x,∆y}  using training samples in the leaf node. When a test sample reaches one leaf node of each tree, regression vectors are predicted by averaging all individual trees in the RF. Because one layer is composed of r-regression forests, the final displacement vector of the *l*th layer $∆d^{l}=\left\{∆x^{l},∆y^{l}\right\}$ is recomputed based on the average output of all RFs.

The proposed CCD-RF structure consists of N layers, where each layer is progressively refined regarding the pupil location in a layer-by-layer manner, as shown in Figure 1b. The first layer estimates the coarse pupil position; however, as the model moves backwards, the layer needs to estimate the fine pupil position. Therefore, not all layers have the same number of RFs; however, as the layers move backwards, the number of RFs gradually increases. Experiments on the optimal layer construction for CCD-RF are described in Section 5.1.

### Pupil Tracking Based on Consistency Check

Although CCD-RF accurately predicts the pupil positions, a false positioning of the pupil center is inevitable owing to a prediction error and/or the presence of some unusual face or eye variations, such as changes in the facial pose changes, occlusions by hair or glasses, or a closed eye. To accurately track the pupil in image sequences while excluding falsely detected pupil positions, eye particle positions (which number three in this study) are propagated by interpolating the predicted pupil position and white Gaussian noise. Then, to maintain an accurate pupil tracking, we check the association between the *j*th eye particle in the current frame $p_{j}\left(t\right)\;$ and the previous pupil position p(t−1) using the $L_{2}\;$ matching function. We then rearrange the final pupil position to one of the eye particles that has the best matching score. This process is conducted separately in the left and right eyes, and matching is not applied if the eyes are closed.

## 5. Experiments

In this section, we demonstrate the excellent performance of the proposed method in terms of accuracy, model size, and energy efficiency by providing a detailed experimental analysis through comparative experiments conducted between the proposed method and previous state-of-the-art approaches using two datasets, namely, GI4E [25] and BioID [26], which have been widely applied in studies on pupil localization.

The GI4E dataset is composed of 1236 color images with a pixel resolution of 800 × 600 taken from 103 subjects with 12 different gaze directions. This dataset includes various illumination, facial pose changes, and occlusions by hair or glasses. To contain the eye movements, this dataset consists of a sequence image set for each subject. It also provides ground truth files for the location of the left and right pupils.

The BioID dataset includes 1521 gray-level images from 23 different subjects with a pixel resolution of 384 × 286. This dataset is more challenging than GI4E because it includes various changes, such as in the illumination, poses, occlusions by hair or glasses, and positions of the faces in a realistic environment. We can use the ground truth files for the left and right pupil positions.

In general, a dataset with a small amount of data utilizes an n-fold cross validation for a fair evaluation. Because the GI4E and BioID datasets also include relatively a small number of images, we conducted an experiment based on a five-fold cross validation. The proposed method was implemented using Python (Centrum Wiskunde & Informatica, Amsterdam, The Netherlands), and the experiments were conducted using Windows 10 (Microsoft, Redmond, WA, USA) with an Intel i7-770K CPU (Intel, Santa Clara, CA, USA); in addition, because other comparative experiments additionally used an NVIDIA GTX 1080Ti GPU (NVIDIA, Santa Clara, CA, USA), an energy consumption test was conducted in Ubuntu on a laptop with an Intel Core i7-7700 CPU (Intel, Santa Clara, CA, USA) and an NVIDIA GeForce GTX 1070 GPU (NVIDIA, Santa Clara, CA, USA).

For a performance evaluation, we measured the accuracy through an evaluation metric called the maximum normalized error. The maximum normalized error is calculated as follows:

(6) $$e_{d}=\frac{\max\left(d_{L},\;d_{R}\right)}{d_{LR}}$$

where $d_{L}$ and $d_{R}$ are the Euclidean distances between the predicted left and right pupil centers and the actual pupil locations, respectively. In addition, $d_{LR}$ is the Euclidean distance between the ground truth of the left and right pupil positions. As in previous gaze tracking studies, we divided the threshold for $e_{d}$ by {0.025, 0.05, 0.1} and estimate the accuracy at each threshold. Here, $e_{d}\leq 0.1$ and $e_{d}\leq 0.05$ indicate that the predicted positions exist somewhere within the iris and pupil areas, respectively.

### 5.1. Determining Optimal Numbers of Layers of CCD-RF

The number of layers is closely related to the accuracy and processing speed. It is therefore essential to find the optimal number of layers to reduce the computational costs while maintaining the level of accuracy. In this experiment, we attempted to determine the appropriate number of layers for the CCD-RF. In the CCD-RF, to predict the finer positions, the deeper layers must be made up of a larger number of forests and decision trees.

We increase the number of layers from one to five, where each layer has a different number of regression trees: (1) 5 forests with each forest consisting of 10 trees, (2) 10 forests with each forest consisting of 20 trees, (3) 15 forests with each forest consisting of 30 decision trees, (4) 20 forests with each forest consisting of 40 decision trees, and (5) 25 forests with each forest consisting of 50 decision trees.

**Figure 3:**
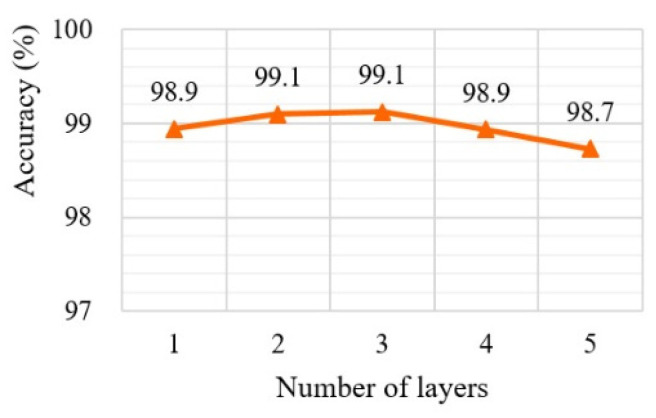
Performance evaluation results according to number of layers with $e_{d}\leq 0.05$ using GI4E dataset.

Figure 3 shows the results of the measurement accuracy for $e_{d}\leq 0.05$ according to the number of layers in CCD-RF using GI4E. As indicated in this figure, the accuracy improves as the number of layers increases from one to three, whereas the accuracy when applying more than three layers decreases.

Because three layers constitute the best coarse-to-fine network, the model with four or more layers is overfitted. Based on this result, we constructed a three-layer CCD-RF to achieve an excellent pupil tracking performance.

### 5.2. Evaluation of Model Simplification

In Section 3, we described the rule distillation for a model simplification to improve the interpretability of the model for a cascade RF architecture. To assess the ripple effect of the model simplification on the proposed CCD-RF, we measured the changes in accuracy and numbers of rules, parameters, and operations according to varying ratios of the rule distillation of 1.0 to 0.5 using the GI4E and BioID datasets.

Table 2 shows the evaluation results in accordance with the rule distillation ratio for the GI4E dataset. In the case of $e_{d}\leq 0.05$ and $e_{d}\leq 0.1$, when we removed 50% of the rules for model simplification, the numbers of parameters and operations significantly reduced to 38% and 5% of the numbers when using all rules during the training process, respectively, while maintaining the level of accuracy. In the case of $e_{d}\leq 0.025$, the decrease in accuracy is larger than that at $e_{d}\leq 0.05$ and $e_{d}\leq 0.1$, although if 30% of the rules are removed, 28% and 4% fewer parameters and operations are required than when all rules are applied, with only a small decrease in accuracy of 1.6%.

Table 3 shows the evaluation results when changing the rule distillation ratios in the BioID dataset. When 40% of the rules are removed, the number of parameters and operations are 27% and 2% lower than when all rules are applied. However, the decrease in accuracy at $e_{d}\leq 0.025$, $e_{d}\leq 0.05$, and $e_{d}\leq 0.1$ was only 1.6%, 2.6%, and 0.7%, respectively. Moreover, we know that both datasets achieve the best accuracy when 10% of the rules are removed, and optimization is achieved by excluding unnecessary rules. Therefore, the rule distillation can enhance the model performance through the removal of unnecessary rules. In addition, the rule distillation in the proposed CCD-RF achieves a model simplification by dramatically reducing the number of parameters and operations. The negligibly small reduction in accuracy caused by the rule distillation is acceptable considering the reduced model size and fewer operations for real-time on-device systems.

### 5.3. Comparison with the State-of-the-Art Methods

To demonstrate the superior pupil tracking performance of the proposed method, we conducted comparative experiments using the state-of-the-art methods mentioned in Section 2. For evaluation with a wider range of studies, we first compared the performance with three major approaches such as traditional machine learning methods with hand-crafted features [12,13,14], cascading method with hand-crafted features [15], GAN-based method [17], CNN-based method [18], CNN with regression framework [27], and specular reflection method [28]. All results of the comparison methods are based on the accuracy from the corresponding papers.

As shown in Table 4, the proposed methods have a high accuracy of about 10% at $e_{d}\leq 0.025$ and $e_{d}\leq 0.05$, and a high performance of about 7% at $e_{d}\leq 0.1\;$ compared to [15] using the cascading method. As for the [17] using GAN, it can be seen that there is little difference between $e_{d}\leq 0.05\;$ and $e_{d}\leq 0.1$. However, when comparing the proposed method with the method of [18], it showed almost similar performance at $e_{d}\leq 0.05$ but about 10% lower performance at $e_{d}\leq 0.025$. This is because the method [18] increased the training data through the GAN and used two heterogeneous CNN models to detect fine location of pupils. It can be seen that the performance of the Bin method [27] or Ahmed method [28] is similar to that of the proposed method at $e_{d}\leq 0.1$, but the accuracy is lower than the proposed method by 2.4–3.7% at $e_{d}\leq 0.05$.

Choi et al. [18] showed the best performance in the GI4E dataset, but in terms of processing, it showed 28 ms in the GTX 1070 GPU environment. In contrast, our method (rule ratio 0.9) achieves a fast processing time of 40 ms in an i7-770K CPU environment without a GPU, which means that an accurate and fast pupil tracking can be conducted on an embedded system.

In terms of the results of Table 5, our proposed method achieved better results at all values of $e_{d}$ than the comparison methods, including [18], for the challenging low-resolution BioID dataset. Bin method [27] showed 0.6% higher accuracy than the proposed method (1.0) at $e_{d}\leq 0.1$, but there was no significant difference. Rather, at $e_{d}\leq 0.05$, which is more precise, the performance was lower by 1.9% than the proposed method (1.0). In particular, the proposed method with 20% rule removed showed better performance than 100% rule in $e_{d}\leq 0.025$ requiring higher precision. This shows that relatively less important rules were effectively removed through rule distillation. From the results of Table 4 and Table 5, we can see that the proposed rule distillation method achieves a certain level of performance even with some removed rules regardless of the resolution of the input image.

Our goal in this study is to develop a pupil tracking algorithm that works on low-spec devices. For this, the proposed CCD-RF should have the proper balance of power, model size, and energy efficiency, while maintaining a reasonable performance on low-spec systems. Therefore, through comparative experiments with a CNN-based architecture, which has recently shown an outstanding performance in pupil tracking, we tried to confirm that the proposed method has a good balance based on three factors. Therefore, as the third experiment, we measured the accuracy, processing time, and numbers of parameters with operations using the general CNN-based ResNet18, ResNet50 [29], and VGG16 [30], as well as CNN-based compression algorithms such as MobileNetV3 [31], using the GI4E and BioID dataset. During this experiment, a model with 10% of the rules removed from the original CCD-RF was used. As shown in Table 6 using the GI4E dataset, the proposed method achieves a similar or better accuracy than the four CNN models at $e_{d}\leq 0.05$ and $e_{d}\leq 0.1$. Although our method has a slightly lower accuracy at $e_{d}\leq 0.025$ in case of ResNet18 of GI4E, it requires extremely smaller specifications, and the numbers of parameters and operations of the proposed method are 14- and 2827-times lower than those of ResNet18, which achieves the highest level of accuracy. For a more objective analysis, experiments were conducted on the BioID dataset with lower image resolution, and it was confirmed that the proposed method shows better performance than ResNet18 in terms of $e_{d}\leq 0.025$ and $e_{d}\leq 0.05$ as shown in Table 7. When analyzing the results of the two experiments, it can be seen that the proposed method is similar to or better than the ResNet-based methods, and in particular, the performance is maintained without deterioration even when some portion of rules are removed or the image resolution is low. In addition, our lightweight CCD-RF achieves the fastest processing speed on a CPU compared with the CNN models applied on a GPU, as shown in Table 6 and Table 7. The lightweight CCD-RF is much faster than the CNN models when considering that no GPU is used.

### 5.4. Energy Efficiency

Hu et al. [32] proposed leakage detection model based on density-based spatial clustering of applications with noise and multiscale fully convolutional networks to manage the water loss. Similar to [32], we conducted a comparative experiment to verify that the proposed method manages the energy consumption efficiently. The energy consumption can be measured using a discharging laptop battery and then utilizing the powerstat utility on a laptop environment.

**Figure 4:**
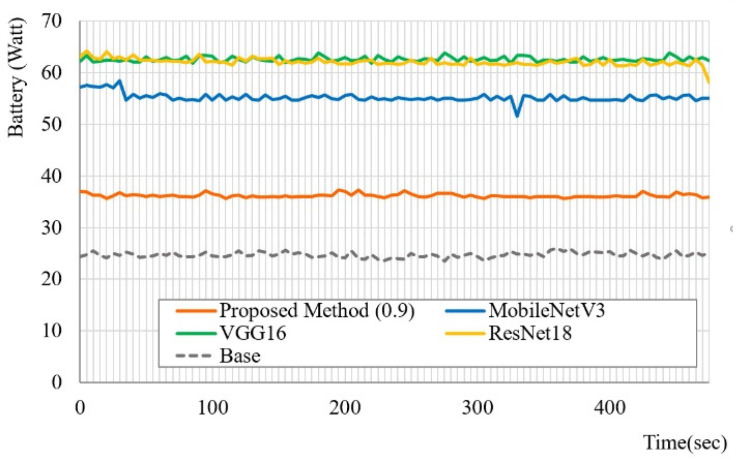
Comparison of battery discharge between our proposed method and CNN-based algorithms with GI4E dataset. “Base” is basically the amount of battery consumed by the OS and other basic utilities.

Figure 4 shows a graph of the battery discharge collected every 5 s for an 8 min period. The proposed method (0.9) used only 90% of the rules from the original CCD-RF model. “Base” in Figure 4 is basically the amount of battery consumed by the OS and other basic utilities. The ResNet50 model was excluded from this experiment because we were unable to measure its performance properly owing to the limited H/W resources available because of the excessive energy consumption. As expected, the proposed method showed the lowest amount of battery consumption compared with the CNN-based models, achieving 1.5-times less battery consumption than MobileNetV3 in particular, which is a popular CNN compressed model. VGG16 has about three times as many operations as ResNet18, but it is faster than ResNet18 due to its simple network structure and shows almost the same battery consumption rate. MobileNetV3 had some fluctuation in the battery consumption rate in a certain time period, but overall, the four methods used in the experiment showed a stable battery consumption rate over time.

The results show that the proposed model can be used to develop a low-power, real-time algorithm that can be interpretable and energy-efficient through the use of the lightweight CCD-RF. Moreover, in terms of accuracy, memory, and energy consumption, the proposed gaze tracking method outperforms recent approaches and can be optimized for various types of embedded systems.

**Figure 5:**
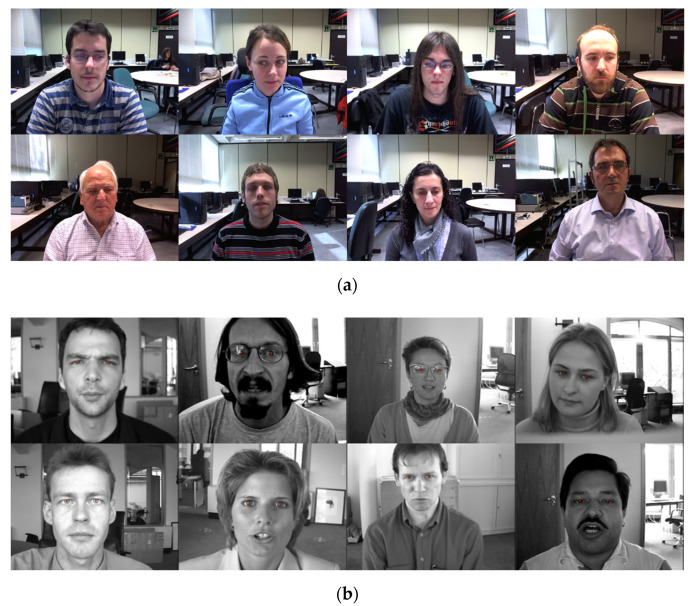
The results of detected and tracked of pupil center by applying the proposed lightweight CCD-R and consistency check to (**a**) GI4E and (**b**) BioID.

Figure 5 shows the qualitative examples of the proposed lightweight CCD-RF. As shown in Figure 5, the proposed approached can be confirmed that the pupil position is effectively tracked even for various positions of the pupil, occlusion, various lighting environments, and glasses.

## 6. Conclusions

In this paper, an energy efficient pupil tracking method developed using a rule distillation and simplification of a black-box CCD-RF was proposed. A model simplification was achieved by applying a modified sequential covering to a fully trained CCD-RF model to induce rules covering each RF, and to distillate low-importance rules according to the feature importance during the process of reconstructing a rule set. Unlike other existing DNN-based model compression techniques, the rule distillation proves that the performance is not degraded even if numerous rules and parameters are eliminated. The proposed pupil tracking model not only consumes much less energy than other DNN-based methods, it also requires less computing power to enable a real-time operation on a CPU as described in Figure 1d. However, the proposed method still requires the distillation of overlapped features, trivial rules, and unnecessary parameters; thus, it cannot be defined as a complete white-box model. A future study will focus on considerably reducing the rule redundancy through a hierarchical rule importance sharing approach using a feature correlation analysis.

## Figures and Tables

**Table 1:** An example of a rule set reconfiguration with the proposed rule distillation.

Initial Rules	Reconfigured Rules
**PLACEHOLDER:** {cumulated contribution} **IF** (feature, condition) AND.. **THEN** {values}	
**1**:{***0.0021***}**IF**(*x_280_*>0.267)and(*x*_261_>0.268)and(*x*_293_>0.139)and(*x*_105_>0.22)**THEN**{0.002, −0.002} **2**:{***0.0029***}**IF**(*x*_280_>0.267)and(*x*_261_>0.268)and(*x*_277_≤0.234)and(*x*_308_≤0.354)and(*x*_251_≤0.226)**THEN**{−0.001,−0.003} **3**:{***0.0004***}**IF**(*x*_280_>0.267)and(*x*_262_>0.426)and(*x*_281_>0.324)and(*x*_354_>0.364)and(*x*_107_≤0.363)**THEN{**0.001, −0.001} …	**1**:{***0.0029***}**IF**(*x*_280_>0.267)and(*x*_261_>0.268)and(*x*_277_≤0.234)and(*x*_308_≤0.354)and(*x*_251_≤0.226)**THEN**{−0.001, −0.003} **2**:{***0.0021***}**IF**(*x_280_*>0.267)and(*x*_261_>0.268)and(*x*_293_>0.139)and(*x*_105_>0.22)**THEN**{0.002, −0.002} **3**:{***0.0004***}**IF**(*x*_280_>0.267)and(*x*_262_>0.426)and(*x*_281_>0.324)and(*x*_354_>0.364)and(*x*_107_≤0.363)**THEN**{0.001, −0.001} …

**Table 2:** Performance evaluation results in terms of accuracy and numbers of rules, parameters, and operations of the proposed method according to changes in the rule distillation when using the GI4E dataset.

Rule Ratio	Accuracy (%)			# of Rules (M)	# of Paramammeters (M)	# of Operations (M)
	$e_{d}\;\leq \;0.025$	$e_{d}\;\leq \;0.05$	$e_{d}\;\leq \;0.1$			
1.0	79.1	99.1	99.9	0.0700	0.8323	0.0080
0.9	79.5	99.3	99.9	0.0637	0.7908	0.0079
0.8	79.1	99.2	99.9	0.0565	0.7336	0.0079
0.7	77.5	99.3	99.9	0.0496	0.6726	0.0078
0.6	75.9	99.3	99.9	0.0425	0.6019	0.0077
0.5	73.4	99.3	99.9	0.0353	0.5198	0.0076

**Table 3:** Performance evaluation results in terms of accuracy and numbers of rules, parameters, and operations of the proposed method according to changes in the rule distillation when using the BioID dataset.

Rule Ratio	Accuracy (%)			# of Rules (M)	# of Parameters (M)	# of Operations (M)
	$e_{d}\;\leq \;0.025$	$e_{d}\;\leq \;0.05$	$e_{d}\;\leq \;0.1$			
1.0	59.4	92.7	99.0	0.2061	2.4641	0.0126
0.9	61.4	93.4	98.7	0.1863	2.3498	0.0126
0.8	62.0	92.7	98.7	0.1656	2.1842	0.0125
0.7	59.7	92.2	98.7	0.1450	2.0055	0.0125
0.6	57.8	90.1	98.3	0.1243	1.8095	0.0124
0.5	55.1	88.1	98.7	0.1035	1.5987	0.0122

**Table 4:** Comparison of the accuracy with state-of-the-art methods using the GI4E dataset.

Methods	Accuracy (%)		
	$e_{d}\;\leq \;0.025$	$e_{d}\;\leq \;0.05$	$e_{d}\;\leq \;0.1$
Zhang et al. [12]	-	97.9	99.6
Xia et al. [13]	-	98.5	99.9
George et al. [15]	69.1	89.3	92.3
Gou et al. [17]	-	98.3	99.8
Choi et al. [18]	90.4	99.6	-
Bin [27]	-	95.4	99.6
Ahmed [28]	-	96.7	98.7
Proposed Method (1.0)	79.1	99.1	99.9
Proposed Method (0.9)	79.5	99.3	99.9
Proposed Method (0.8)	79.1	99.2	99.9

**Table 5:** Comparison of the accuracy with state-of-the-art methods using the BioID dataset *.

Methods	Accuracy (%)		
	$e_{d}\;\leq \;0.025$	$e_{d}\;\leq \;0.05$	$e_{d}\;\leq \;0.1$
Zhang et al. [12]	-	85.7	93.7
Xia et al. [13]	-	88.1	98.8
Ince et al. [14]	-	89.0	97.5
George et al. [15]	-	85.1	94.3
Gou et al. [17]	-	92.3	99.1
Choi et al. [18]	60.0	93.3	-
Bin [27]	-	90.8	99.6
Ahmed [28]	-	91.6	98.6
Proposed Method (1.0)	59.4	92.7	99.0
Proposed Method (0.9)	61.4	93.4	98.7
Proposed Method (0.8)	62.0	92.7	98.7

* Because the source codes of the comparison methods were not open, we referred the accuracy from the papers measured using GI4E and BioID dataset.

**Table 6:** Comparison of accuracy, processing time, and numbers of parameters and operations with convolutional neural network (CNN)-based models using GI4E dataset. The proposed method removes 10% of the rules through a rule distillation from the original CCD-RF model.

Methods	Accuracy (%)			# of Parameters (M)	# of Operations (M)	Processing Time (ms)
	$e_{d}\;\leq \;0.025$	$e_{d}\;\leq \;0.05$	$e_{d}\;\leq \;0.1$			
ResNet50 [29]	64.2	96.7	99.6	38.08	76.05	54.8(GPU)
VGG16 [30]	10.8	63.3	98.8	18.89	67.02	61.6(GPU)
ResNet18 [29]	82.0	98.7	100	11.17	22.33	43.0(GPU)
MobileNetV3 [31]	26.2	68.6	98.3	1.52	3.02	47.6(GPU)
Proposed Method (0.9)	79.5	99.3	99.9	0.7908	0.0079	40.2(CPU)

**Table 7:** Comparison of accuracy, processing time, and numbers of parameters and operations with CNN-based models using BioID dataset. The proposed method removes 10% of the rules through a rule distillation from the original CCD-RF model.

Methods	Accuracy (%)			# of Parameters (M)	# of Operations (M)	Processing Time (ms)
	$e_{d}\;\leq \;0.025$	$e_{d}\;\leq \;0.05$	$e_{d}\;\leq \;0.1$			
ResNet50 [29]	62.8	92.4	98.9	38.08	76.05	54.2(GPU)
VGG16 [30]	21.3	69.9	95.2	18.89	67.02	42.2(GPU)
ResNet18 [29]	54.2	86.3	98.7	11.17	22.33	39.3(GPU)
MobileNetV3 [31]	40.1	83.1	96.5	1.52	3.02	41.2(GPU)
Proposed Method (0.9)	61.4	93.4	98.7	0.9435	0.0088	40.2(CPU)
